# Computational characterization of inhaled droplet transport to the nasopharynx

**DOI:** 10.1038/s41598-021-85765-7

**Published:** 2021-03-23

**Authors:** Saikat Basu

**Affiliations:** 1 Department of Mechanical Engineering, South Dakota State University, Brookings, SD 57007 USA; 2 Department of Otolaryngology / Head and Neck Surgery, University of North Carolina School of Medicine, Chapel Hill, NC 27599 USA

**Keywords:** Translational research, Fluid dynamics, Biomedical engineering, Respiratory tract diseases

## Abstract

How human respiratory physiology and the transport phenomena associated with inhaled airflow in the upper airway proceed to impact transmission of SARS-CoV-2, leading to the initial infection, stays an open question. An answer can help determine the susceptibility of an individual on exposure to a COVID-2019 carrier and can also provide a preliminary projection of the still-unknown infectious dose for the disease. Computational fluid mechanics enabled tracking of respiratory transport in medical imaging-based anatomic domains shows that the regional deposition of virus-laden inhaled droplets at the initial nasopharyngeal infection site peaks for the droplet size range of approximately 2.5–19 $\upmu$. Through integrating the numerical findings on inhaled transmission with sputum assessment data from hospitalized COVID-19 patients and earlier measurements of ejecta size distribution generated during regular speech, this study further reveals that the number of virions that may go on to establish the SARS-CoV-2 infection in a subject could merely be in the order of hundreds.

## Introduction

Severe acute respiratory syndrome coronavirus 2 (SARS-CoV-2) has been identified as the causative agent for coronavirus disease 2019 (COVID-19), that has inflicted a global pandemic with nearly 114 million confirmed infections and over 2.5 million deaths worldwide, as of late-February 2021; for details, see^1^.

As is well-known by now, transmission of respiratory infections such as COVID-19 occurs through carriage of pathogens via droplets of different sizes produced during sneezing, coughing, singing, normal speech, and even, breathing^2^. Accordingly, the means of person-to-person infection are projected to be three-way^3^: (a) inhalation of virus-laden droplets emitted by an infected individual at close-range; (b) inhalation of vaporized droplet nuclei that can float in air for hours; and (c) contaminating the respiratory mucosa through physical contact to external surfaces (*fomites*) with droplet deposits sitting on them. While (a) is valid for short-distance exposures to the COVID-19 carrier, transmission through modes (b) and (c) can happen over larger distances and longer time scales. However, clustering trends of infection spread (e.g. in industrial units^4^, in closed groups^5^, and inside households^6^) suggest that close-range exposures, through inhalation of respiratory ejecta from infected individuals, is a critical determinant in worsening of the pandemic. A follow-up question might be—*what entails an exposure?* A key component therein are the respiratory droplet sizes one is exposed to. Coughing and sneezing typically generate droplets with length-scales of $\mathcal {O}(10^2)$ to $\mathcal {O}(10^3)$ $\upmu$, while oral droplets ejected during normal speaking can range over $\sim$ 0.1–500 $\upmu$^3,7^. Some of the competing environmental effects determining the fate of these droplets are the ambient temperature and humidity (e.g. low relative humidity induces fast evaporation and shrinkage of the droplets), along with the size of the droplet that controls its inertia and the gravitational force acting on it. While smaller droplets would stay airborne for longer, the larger droplets tend to fall fast ballistically; with the critical size for this transition being in the vicinity of 100 $\upmu$^8,9^. Of note here, this study does not insist on any nomenclatural distinction between “aerosols” and “droplets”, owing to ambiguities^10^ in common perception, and simply refers to all expiratory liquid particulates as *droplets*.

**Figure 1:**
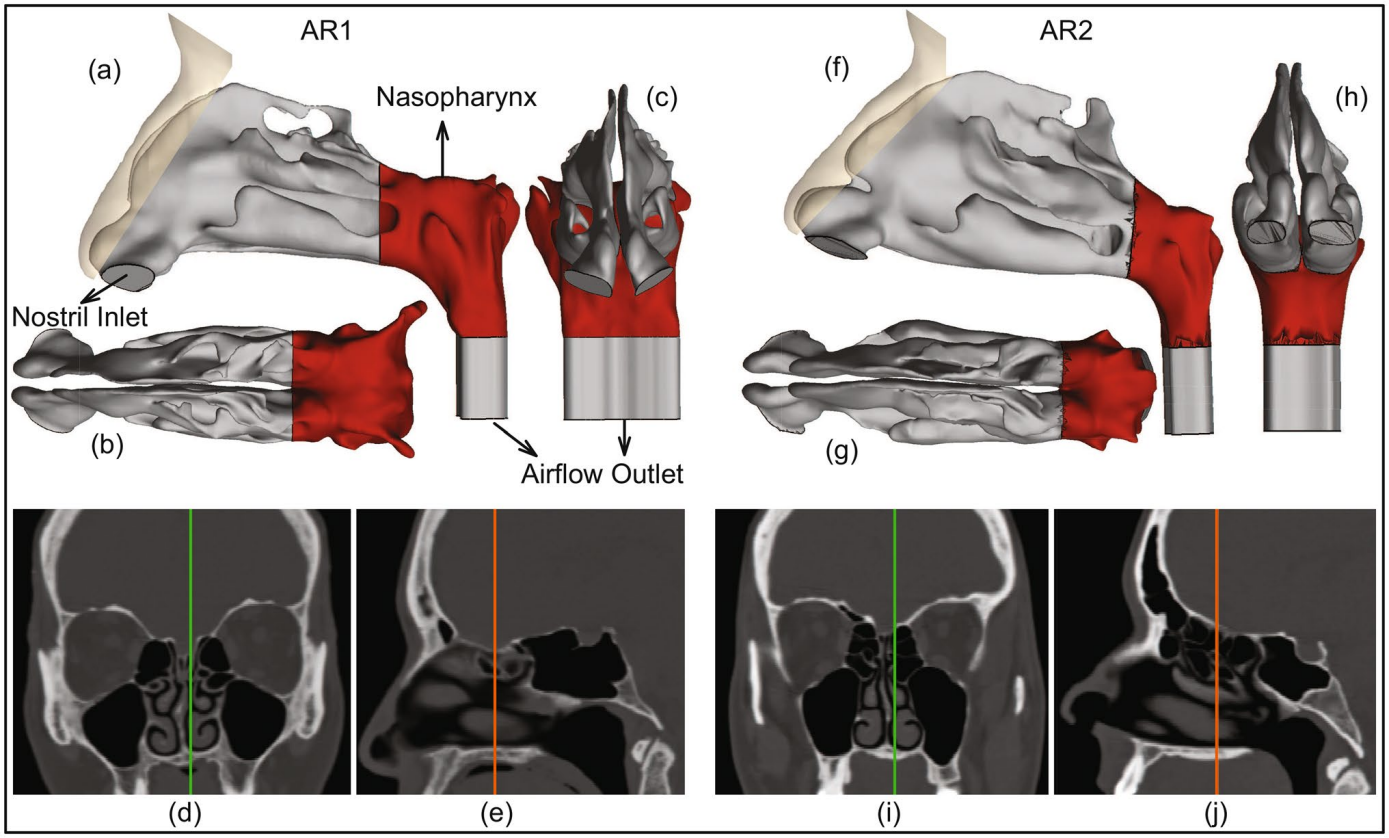
Anatomically realistic nasal airway geometries:  (**a**–**c**) respectively depict the sagittal, axial, and coronal views of the computed tomography (CT) based nasal domain in anatomic reconstruction 1 (AR1). (**d**,**e**) Representative CT slices for AR1. Therein, the green line in (**d**) corresponds to the location of the sagittal section shown in (**e**); the orange line in (**e**) corresponds to the location of the coronal section shown in (**d**). (**f**–**h**) Respectively depict the sagittal, axial, and coronal views of the CT-based nasal domain in anatomic reconstruction 2 (AR2). (**i**,**j**) Representative CT slices for AR2. Therein, the green line in (**i**) corresponds to the location of the sagittal section shown in (**j**); the orange line in (**j**) corresponds to the location of the coronal section shown in (**i**). The digital reconstructions only consider the main nasal cavity and leave out the sinus appendages. Nasopharynx is marked in red on the top row graphics. Visuals in (**a**–**c**), (**f**–**h**) are generated using the software ICEM CFD 15.0 (ANSYS Inc., Canonsburg, Pennsylvania; link to software homepage). The scans on (**d**,**e**) and (**i**,**j**) are extracted from the image processing software Mimics Research 18.0 (Materialise, Plymouth, Michigan; link to software homepage).

For tracking what range of virus-bearing droplet sizes might be more potent for SARS-CoV-2 transmission and to eventually induce infection, it is crucial that we identify the initial infection sites. At least two 2020 studies^11,12^ reveal a striking pattern of relatively high SARS-CoV-2 infectivity in ciliated epithelial cells along the nasal passage lining in the upper airway, to less infectivity in cells lining the throat and bronchia, and finally to relatively low infectivity at the lung cells. Such viral tropism is decidedly governed through angiotensin-converting enzyme 2 (ACE2), which is a single-pass type I membrane protein and is the surface receptor that the virus utilizes to intrude into cells. ACE2 is abundant on ciliated epithelial cells, but is relatively scarce on the surface of the lower airway cells. While these findings are for in vitro samples, deposition of virus-laden droplets along the anterior nasal airway might not be so effective as to launch an infection despite the presence of ciliated cells, since the mucus layer provides some protection against virus invasion and infection^3^. This sets up *nasopharynx* (i.e. the region in the upper airway posterior to the septum and comprising the superior portion of the pharynx; for reference, see Fig. 1) as the main *initial* infection site; it acts as the seeding zone for subsequent infection of the lower airway, mediated by the aspiration of virus-laden boluses of nasopharyngeal fluids^11,13–15^. The *ansatz* steering the identification of nasopharynx, as the dominant initial site of infection during SARS-CoV-2 pathogenesis, is supported by the efficacy^16^ of nasopharyngeal swab testing for COVID-19 diagnosis, when compared to oropharyngeal swabs. So at this point, a valid question to ask would be: *what are the dominant inhaled droplet sizes that are making their way to the nasopharynx?*

Respiratory droplets, on being expelled, typically lose water and shrink;—the extent of which partially depends on the fraction of non-volatile constituents present in the droplets, e.g. dehydrated epithelial cell remnants, white blood cells, enzymes, DNA, sugars, electrolytes etc. So, although sputum is composed of 99.5% water; ejected droplets, on dehydration, have a higher density of 1.3 g/mL^17^, which is what has been used for the initial set of droplet tracking simulations here, with the assumption that the non-volatile weight fraction is in the 1–5% range. Such dehydration contracts the expelled droplet diameter to 20–34% of the initial size. Considering 100 $\upmu$ as the critical size prompting ballistic sedimentation, this study tracks inhaled droplet sizes that could still be airborne after dehydration. Hence, the tested size range is 0.1–30 $\upmu$, assuming a conservative dehydration-induced shrunk volume of 30% of the initial size^18^. Choice of the smallest tracked droplet size is dictated by SARS-CoV-2 dimension, which is in between 0.08 and 0.2 $\upmu$, with an average physical diameter of 0.1 $\upmu$^19^.

Next piece in this puzzle involves the breathing parameters. Allometric relations^20^ put the minute inhalation at 18.20 L/min for a 75-kg male and 15.05 L/min for a 75-kg female, for gentle steady breathing while sitting awake. In general, inspiratory rates can stretch over $\sim$ 15 to 85 L/min, based on whether the individual is inhaling gently or breathing in forcefully. This study uses computational fluid dynamics (CFD) in anatomically realistic upper airway geometries to simulate droplet transmission at four different inhalation rates, viz. 15, 30, 55, and 85 L/min; notably these discrete flow rates are also the ones traditionally used^21^ for checking filtration capacities of protective face-coverings and respirators. The flow physics undergo a transition over this range; e.g. 15 L/min through nasal conduits is in laminar regime, the transport mechanism however devolves into turbulence at higher inhalation rates.

## Methods

### Anatomic geometry reconstruction

Computed tomography (CT)-based in silico model generation was accomplished according to relevant guidelines and regulations, with the anatomic geometries being reconstructed from existing de-identified imaging data from two CT-normal subjects. The use of the archived and anonymized medical records was approved with exempt status by the Institutional Review Board of the University of North Carolina (UNC) at Chapel Hill, with the requirement of informed consent being waived for retrospective use of the de-identified scans in computational research. The test subjects include a 61 year-old female (subject for anatomic reconstruction 1, or AR1) and a 37 year-old female (subject for anatomic reconstruction 2, or AR2). In context to the imaging resolution, the CT slices were collected at coronal depth increments of 0.348 mm in AR1’s scans and 0.391 mm in AR2’s scans. The nasal airspaces were extracted from the medical grade scans (see the bottom row in Fig. 1 for representative CT slices) over a delineation range of − 1024 to − 300 Hounsfield units, and was complemented by careful hand-editing of the selected pixels to ensure anatomic accuracy. For this step, the DICOM (Digital Imaging and Communications in Medicine) scans for each subject were imported to the image processing software Mimics Research 18.0 (Materialise, Plymouth, Michigan).

The reconstructed geometries were exported as stereolithography files to ICEM-CFD 15.0 (ANSYS Inc., Canonsburg, Pennsylvania), and then meshed spatially into minute volume elements. Conforming with established mesh refinement-based protocols^22,23^, each computational grid contained more than 4 million unstructured, graded tetrahedral elements (e.g. 4.54 million in AR1, 4.89 million in AR2); along with three prism layers of 0.1-mm thickness at the airway walls, with a height ratio of 1. The nostril inlet planes comprised 3015 elements in AR1 (1395 elements on left nostril plane, 1620 elements on right nostril plane) and 3000 elements in AR2 (1605 on left nostril plane, 1395 on right nostril plane).

### Numerical simulations

The study considers droplet transport for four different inhaled airflow rates, viz. 15, 30, 55, and 85 L/min. The lower flow rate (i.e. 15 L/min) corresponds to comfortable resting breathing, with the viscous-laminar steady-state flow physics model standing in as a close approximation^24–35^. At higher flow rates (extreme values of which may sometimes lead to nasal valve collapse), the shear layer separation from the tortuous walls of the anatomic geometries results in turbulence^36–40^. While accounting for the turbulent characteristics of the ambient airflow, the study averages the droplet deposition percentages from implementation of two distinct categories of numerical schemes, viz. (a) shear stress transport (SST) based k-$\omega$ model, which is a sub-class under Reynolds-averaged Navier Stokes (RANS) schemes that parameterize the action of all turbulent fluctuations on to the mean flow; and (b) Large Eddy Simulation (LES). In this work (see results), the two numerical techniques depict high correlation in terms of droplet deposition at the nasopharynx. However, it should be noted that while the SST k-$\omega$ scheme, a 2-equation eddy-viscosity model, is computationally less expensive; it averages the short time-scale flow artifacts, such as the transient vortices (e.g. the low-pressure Dean vortices that are common in tortuous channels and can act as droplet attractors), and hence the prediction of droplet transport affected by the simulated airflow may at times contain errors. LES is computationally more expensive, it separates the turbulent flow into large-scale and small-scale motions, and accounts for the small fluctuations through a sub-grid scale model (for this study, Kinetic Energy Transport Model was used as the sub-grid scale model^41^). We took the averaged estimates for regional droplet deposition (along the in silico nasal tissue surfaces) from the two schemes, to minimize probable statistical and algorithmic biases.

The computational schemes implemented in the meshed domains employed a segregated solver on ANSYS Fluent 15.0, with SIMPLEC pressure-velocity coupling and second-order upwind spatial discretization. Solution convergence was monitored by minimizing the mass continuity and velocity component residuals, and through stabilizing the mass flow rate and static pressure at the airflow outlets. For the pressure-driven flow solutions: typical convergence run-time in a laminar simulation with 5000 iterations was approximately 5–6 h for 4-processor based parallel computations executed at 4.0 GHz speed. The corresponding run-time for a RANS simulation was $\sim$ 12 h; for an LES computation, it was 4–5 days. Note that for the LES work, the simulated flow interval was 0.5 s for the 30 L/min case, with 0.0002 s as the time-step^42^ and it was 0.25 s for the 55 and 85 L/min flow rates with the time-step at 0.0001 s. In the computations, assumed air density was 1.204 kg/m$^3$ and $1.825\times 10^{-5}$ kg/m s was used as dynamic viscosity of air.

Following set of boundary conditions were enforced during the simulations: (1) zero velocity at the airway-tissue interface i.e. at the walls enclosing the digitized nasal airspace (otherwise commonly referred to as the *no slip* condition), along with “trap” boundary condition for droplets whereby the tracking of a droplet’s transport would cease once it has landed on the walls; (2) zero pressure at nostril planes, which were the pressure-inlet zones in the simulations, with “reflect” boundary condition for droplets to mimic the effect of inhalation on the droplet trajectories if they are about to fall out of the anterior nasal domain; and (3) a negative pressure at the airflow outlet plane, which was the pressure-outlet zone, with “escape” boundary condition for droplets, i.e. allowing for the outgoing droplet trajectories to leave the upper respiratory airspace. Mean inlet-to-outlet pressure gradients were − 9.01 Pa at 15 L/min, − 26.65 Pa at 30 L/min, − 73.73 Pa at 55 L/min, and − 155.93 Pa at 85 L/min. For a reference on the general layout of the anatomic regions, see Fig. 1.

On convergence of the airflow simulations, inhaled droplet dynamics were tracked by Lagrangian-based discrete phase inert particle transport simulations in the ambient airflow; with the localized deposition along the airway walls obtained through numerically integrating transport equations^43^ that consider contribution of the airflow field on the evolution of droplet trajectories, along with the effects for gravity and other body forces such as the Saffman lift force that is exerted by a flow-shear field on small particulates moving transverse to the streamwise direction. Also, the droplet size range is considered large enough to discount Brownian motion effects on their spatial dynamics. Note that the study simulated the transport for 3015 droplets of each size in AR1 and 3000 droplets of each size in AR2, the numbers being same as the number of elements on the nostril inlet planes which were seeded with the to-be-tracked droplets for the droplet transport simulations. For the numerical tracking, the initial mass flow rate of the inert droplets moving normal to the inlet planes into the nasal airspace was required to be non-zero, and was set at $10^{-20}$ kg/s. After the transport simulations, the post-processing of the droplet transmission data along the airway walls provided the regional deposition trends at the nasopharynx.

The numerical methods, discussed and used here, are an extension from one of our recent publications^43^ in this journal. The questions explored in the present study are, of course, very different and new, and the findings can be potentially substantial in our evolving field of knowledge on respiratory pathogen transport, with the current focus being on SARS-CoV-2 transmission mechanisms. The reader should also note that the numeric protocol has been rigorously validated in the earlier publication^43^, through comparing the regional deposition trends along the inner walls of similar in silico nasal anatomic domains to the in vitro spray tests performed in 3D-printed solid replicas of the same reconstructions.

### Assessing the probability for at least 1 virion being embedded on a respiratory ejecta droplet emanating from an infected individual

A recent landmark paper^17^ reports the probability that a 10-$\upmu$ undehydrated droplet, constituted from the respiratory ejecta of an infected individual, will contain at least one SARS-CoV-2 virion is 0.37%. The probability drops to only 0.01%, if the undehydrated droplet diameter is 3 $\upmu$. We attempt in this study to verify their reported data through a simple mathematical model, while at the same time laying down the calculation framework to estimate the number of virions that are directly carried to the nasopharynx by the droplets that could now be inhaled by an exposed individual.

In the oral fluids of hospitalized COVID-19 patients, the mean SARS-CoV-2 RNA load has been measured^14^ at $7.0 \times 10^6$ copies/mL. Imposing a continuum approach and assuming a homogeneous concentration of the virions in the undehydrated respiratory ejecta, 1 m$^3$ of oral ejecta therefore has $7 \times 10^{12}$ virions (given that SARS-CoV-2 is a single-stranded RNA virus^44^). Consequently, an expiratory droplet of diameter $\delta$ (in $\upmu$) would have

1 $$\begin{aligned} \mathcal {N}&= 7\times 10^{12}\times \frac{4\pi }{3}\times \left( \frac{\delta \times 10^{-6}}{2}\right) ^3~\text {virions}. \end{aligned}$$

In other words, to come across 1 virion in undehydrated droplets of diameter $\delta$ (in $\upmu$), one would need to “check”

2 $$\begin{aligned} \mathfrak {n} = \mathcal {N}^{-1}~\text {such droplets}. \end{aligned}$$

Thus, the probability of there being at least 1 virion in an undehydrated droplet of diameter $\delta$ (in $\upmu$) is

3 $$\begin{aligned} \mathcal {P} = \frac{1}{\mathfrak {n}}\times 100\% = f(\delta ). \end{aligned}$$

Plugging in $\delta = 3\,\upmu$ and $10\,\upmu$ in Eqs. (1)–(3) respectively generates $\mathcal {P} = 0.01\%$ and $0.37\%$, thereby matching prior findings^17^ and validating the above mathematical idealization. We have used Eq. (1) (see next, “Estimating the number of virions transmitted to the nasopharynx”) for further quantification of the number of virions carried by the droplets that undergo nasopharyngeal deposition.

### Estimating the number of virions transmitted to the nasopharynx

**Figure 2:**
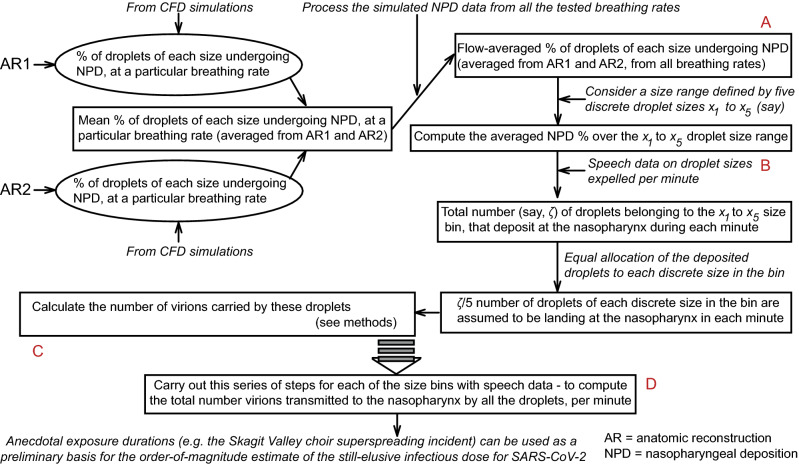
Logic diagram:  for each tested breathing rate, the simulations generate the percentage of droplets of each size that undergo nasopharyngeal deposition (NPD) in AR1 and AR2. From that data, we compute the mean NPD for each droplet size, at each inhalation rate. Next, considering the mean NPD for all tested airflow rates, we compute the flow-averaged NPD for each droplet size. The NPD% rate for each droplet size is then linked to known data on droplet sizes emitted during regular speech^7^, to figure out how many of those droplets would land at the exposed individual’s nasopharynx. We then compute the virion loading, as a function of the droplet sizes—see “Assessing the probability for at least 1 virion being embedded on a respiratory ejecta droplet emanating from an infected individual” and “Estimating the number of virions transmitted to the nasopharynx” for details of the mathematical framework, to quantify the number of virions (say, $\beta$) that are transmitted by the droplets to the nasopharynx in each unit of time. Finally, to obtain an order-of-magnitude estimate of the infectious dose, we multiply $\beta$ with the exposure time reported during a known superspreading incident^5^. Alphabets A–D in the above schematic respectively correspond to the computed outputs in Panels **A**–**D** of Fig. 5. Note that while using the previously reported^7^ ejecta size distribution, the study divides up the percentages for each size bin (i.e. 0–5, 5–10, 10–15 $\upmu$ etc.) uniformly and apportions them to the discrete droplet sizes (belonging to the same size bin) that are tracked (see horizontal axis between the NPD heat-maps in Figs. 3 and 4), to estimate how many droplets of each size would be ejected by the carrier during unit time.

The logic diagram in Fig. 2 lays out the *modus operandi* for the estimation of the number of virions that are carried by the inhaled droplets to the nasopharynx. In essence, we use known data on the distribution of dehydrated droplet sizes that are expelled during regular speaking^7^. The CFD simulations are then used to quantify what percentage of the droplets, purportedly expelled by a COVID-positive patient in proximity, will land at the nasopharynx of the exposed individual. We obtain both the number and sizes of the droplets that undergo nasopharyngeal deposition, and then use Eq. (1), to quantify the approximate number of virions that should be transmitted by those droplets. Note that at this point, we do account for the environmental dehydration, e.g. an expelled droplet of diameter $\delta$ $\upmu$ would undergo dehydration and with mean shrinkage^17^, transform into a droplet of diameter $\mathbb {D} = 0.30 \times \delta$ (in $\upmu$) before being inhaled by an exposed subject, by losing bulk of its volatile water components, and yet would carry the same viral load that was embedded in its initial $\delta$-$\upmu$ size.

**Figure 3:**
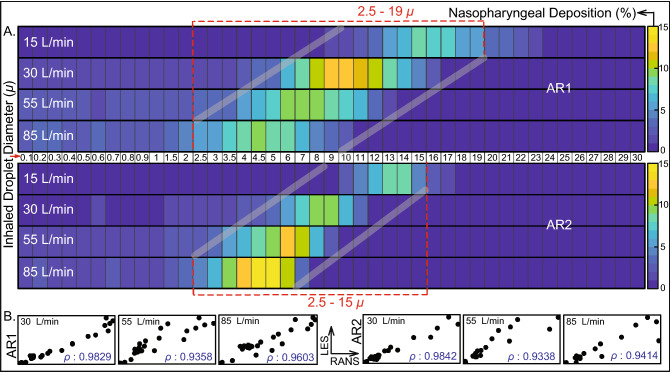
Computed trends of inhaled droplet transport to the nasopharynx:  (**A**) Visuals of heat-maps for inspiratory transmission trends in AR1 (top map) and AR2 (bottom map), showing the percentage of droplets of each size undergoing nasopharyngeal deposition (NPD). The droplets are assumed to undergo environmental dehydration before being inhaled; hence the droplet density is 1.3 g/mL. Data for different inhaled airflow rates are arranged along separate rows. Tracked droplet sizes are along the horizontal axis (positioned between the two heat-maps for AR1 and AR2). NPD peaks for droplets sized between 2.5 and 19 $\upmu$ in AR1 and 2.5 and 15 $\upmu$ in AR2. (**B**) The correlation between RANS-based SST k-$\omega$ and LES results for the higher airflow rates i.e. 30, 55, and 85 L/min; therein the first three frames (bottom-left) are for AR1, the other three frames (bottom-right) correspond to data for AR2. The frames are on an aspect ratio of 0.5; $\rho$ represents the Pearson’s correlation coefficient. Heat-maps in (**A**) are generated by post-processing the simulated data on MATLAB R2020a (MathWorks, Natick, Massachusetts; link to software homepage). The correlation plots in (**B**) are generated on Mathematica 12.0 (Wolfram Research, Champaign, Illinois; link to software homepage).

**Figure 4:**
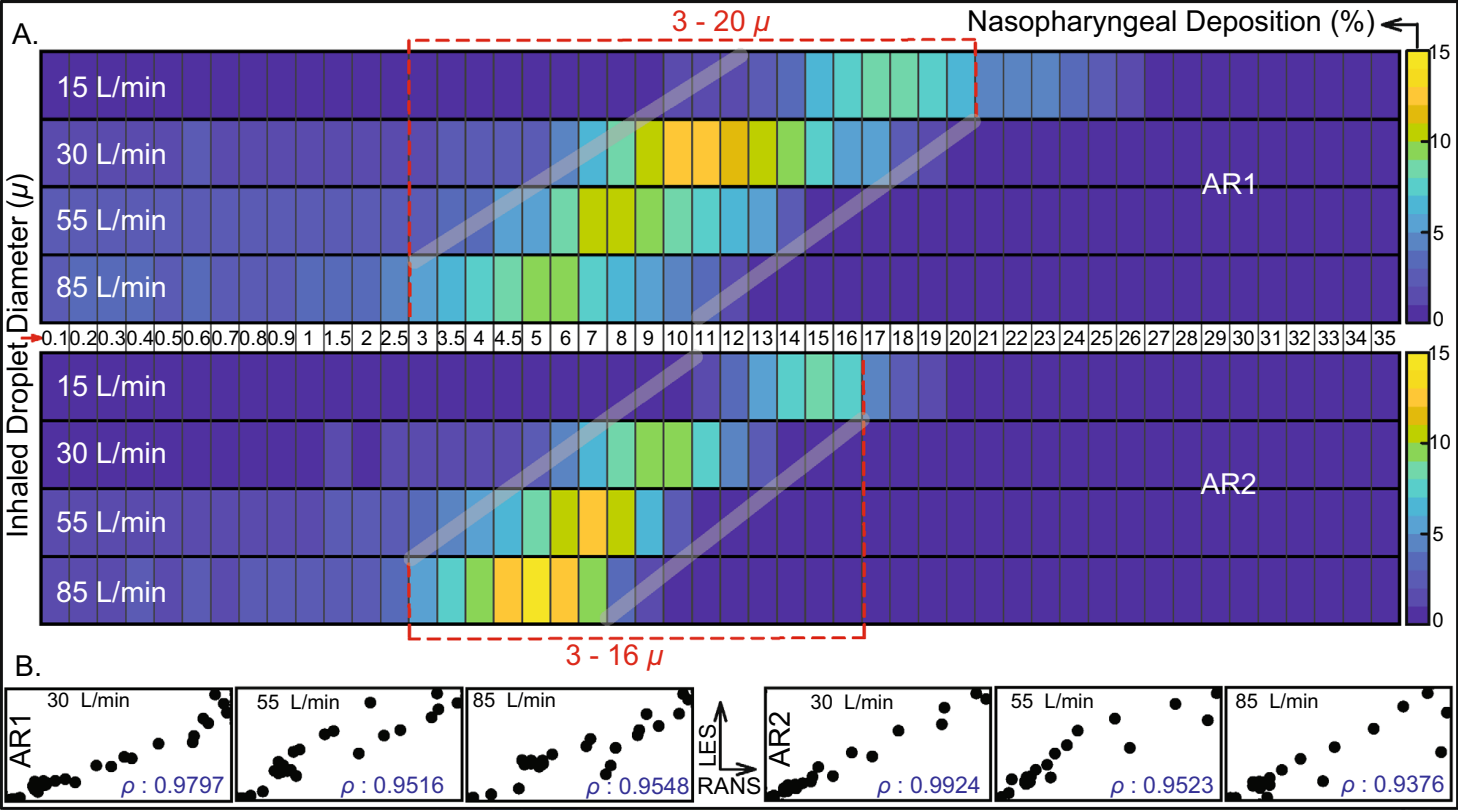
Hazardous droplet size range, when there is minimal or no dehydration:  (**A**)  Visuals of heat-maps for inspiratory transmission trends in AR1 (top map) and AR2 (bottom map), showing the percentage of droplets of each size undergoing nasopharyngeal deposition (NPD). Without any dehydration, the droplet density is assumed to be 1.0 g/mL. Data for different inhaled airflow rates are arranged along separate rows. Tracked droplet sizes are along the horizontal axis (positioned between the two heat-maps for AR1 and AR2). NPD peaks for droplets sized between 3 and 20 $\upmu$ in AR1 and 3 and 16 $\upmu$ in AR2. (**C**) The correlation between RANS-based SST k-$\omega$ and LES results for the higher airflow rates i.e. 30, 55, and 85 L/min; therein the first three frames (bottom-left) are for AR1, the other three frames (bottom-right) correspond to data for AR2. The frames are on an aspect ratio of 0.5; $\rho$ represents the Pearson’s correlation coefficient. Heat-maps in (**A**) are generated by post-processing the simulated data on MATLAB R2020a (MathWorks, Natick, Massachusetts; link to software homepage). The correlation plots in (**B**) are generated on Mathematica 12.0 (Wolfram Research, Champaign, Illinois; link to software homepage).

**Figure 5:**
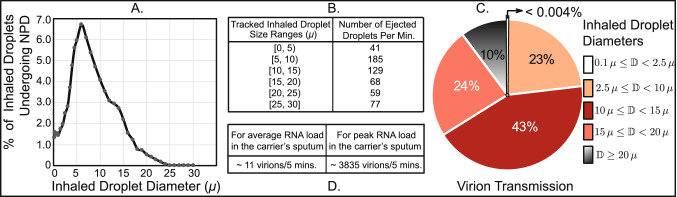
Quantifying virion transmission to the nasopharynx:  (**A**) Graphical representation of the flow-averaged data on the percentage of droplets of each size undergoing deposition at the nasopharynx. (**B**) Distribution of droplet sizes ejected each minute during normal speaking, the numbers are calculated from earlier studies on expelled droplet tracking with food coloring^7^; the reported size distribution is for dehydrated droplets. Also note that the referenced article^7^ described the size bin limits as $\alpha - \beta$ (in $\upmu$). For consistency, we typically interpreted that as droplet sizes (in $\upmu$) that are $\ge \alpha$ and $< \beta$; and in this graphic, such a droplet size bin range is represented by $[\alpha ,\beta )$, conforming to *set theory* notations. (**C**) Pie diagram showing which droplet sizes are dominant contributors for virion transmission at the nasopharynx, for ejecta size distribution as in (**B**). Symbol $\mathbb {D}$ is the inhaled droplet diameter. The numbers assume that the droplets have undergone dehydration before being inhaled into the nasal airspace. (**D**) Estimated number of virions that are deposited at the susceptible individual’s nasopharynx via dehydrated inhaled droplets, during close-range exposure to a COVID-19 carrier for 5 min. Outputs in (**A**–**D**) in the above graphic respectively correspond to the steps marked by labels **A**–**D** in Fig. 2. The plots on (**A**,**C**) are generated using Microsoft Excel 365; link to software homepage.

### Preprint

A preprint version of this manuscript has been screened for content and posted on medRxiv. https://doi.org/10.1101/2020.07.27.20162362.

## Results

### Droplet size range that targets the nasopharynx

The overall droplet size range of 2.5–19 $\upmu$ (in AR1: 2.5–19 $\upmu$, in AR2: 2.5–15 $\upmu$) registers the peak, in terms of the percentage of dehydrated droplets of each size that are deposited at the nasopharynx of an exposed individual. The range is determined by a cut-off of at least 5% deposition for around 3000 tracked droplets (viz. 3015 in AR1, 3000 in AR2) of each size. Panel A in Fig. 3 displays the heat-maps for nasopharyngeal deposition (NPD) for different droplet sizes, during inhalation at the four tested airflow rates. The discrete droplet sizes, that were tracked, have been marked along the horizontal axis of the heat-maps. The patch bounded by the grey lines can, in fact, be a *definitive graphical technique* to delineate the hazardous droplet size range for various airborne transmissions.

#### What if there were less or no environmental dehydration?

Findings in “Droplet size range that targets the nasopharynx” assume that the post-dehydration density of the respiratory droplets (expelled by the carrier and now being inhaled by the exposed individual) is at 1.3 g/mL. If, on the contrary, there is little or no dehydration, the ejected droplet density would remain approximately at $\sim$ 1 g/mL, since pure saliva contains 99.5% water while exiting the salivary glands. With such material density, the inhaled droplet size range for peak NPD upscales to 3–20 $\upmu$, as depicted in Fig. 4. Note that for these lighter droplets, the tested droplet size range was expanded to 0.1–35 $\upmu$ (instead of 0.1–30 $\upmu$, as used for the dehydrated heavier droplets). The somewhat lighter droplets can now penetrate further into the intranasal airspace, the transport process being aided by the inspiratory streamlines. The underlying physical principle can be assessed in terms of the non-dimensional Stokes number and the inertial motion of the droplets.

The Stokes number (Stk) can be mathematized as^45^

4 $$\begin{aligned} \text {Stk} = \frac{U \, \xi _{\mathbb {D}} \, \mathbb {D}^2 \, C_c}{18 \, \mu \, d}. \end{aligned}$$

Here *U* is flow rate divided by flux area, $\xi _{\mathbb {D}}$ is the material density of the inhaled droplets, $C_c$ is the Cunningham slip correction factor, $\upmu$ is the dynamic viscosity of the ambient medium i.e. air, and *d* is the characteristic diameter of the flux cross-section. All other flow and morphological parameters staying the same, it is straightforward to show from Eq. (4) that

5 $$\begin{aligned} \frac{\xi _{\mathbb {D}_1}}{\xi _{\mathbb {D}_2}} = \left( \frac{\mathbb {D}_2}{\mathbb {D}_1}\right) ^2, \end{aligned}$$

where $(\xi _{\mathbb {D}_i},\mathbb {D}_i)$ for $i = 1, 2$ are two different droplet density and droplet size pairings. In this study, we have seen that for say, $\xi _{\mathbb {D}_1} = 1.3$ g/mL, the droplet size range where NPD peaks is 2.5–19 $\upmu$. Let us now use the scaling argument in Eq. (5), to predict the corresponding size range if the droplets do not undergo dehydration, and as such $\xi _{\mathbb {D}_2} = 1.0$ g/mL. If the to-be-predicted size range that would generate peak NPD for the lighter droplets is represented by $\mathbb {D}'_{\text {min}}$ to $\mathbb {D}'_{\text {max}}$ (in $\upmu$), then

6 $$\begin{aligned} \frac{1.3}{1.0} = \left( \frac{\mathbb {D}'_{\text {min}}}{2.5}\right) ^2 \quad \text {and}\quad \frac{1.3}{1.0} = \left( \frac{\mathbb {D}'_{\text {max}}}{19.0}\right) ^2. \end{aligned}$$

This results in $\mathbb {D}'_{\text {min}} = 2.9$ $\upmu$ and $\mathbb {D}'_{\text {max}} = 21.7$ $\upmu$. Despite the simplicity of the scaling analysis, the theoretically predicted range 2.9–21.7 $\upmu$ is reassuringly close to the CFD-based projection of 3–20 $\upmu$.

### Statistical analysis and data interpretation

Consider representatively the simulated intranasal transmission for regular dehydrated droplets. Panel B in Fig. 3 plots the NPD values from RANS (along horizontal axis) and LES (along vertical axis) schemes, implemented for the higher inhalation rates (i.e. 30, 55, and 85 L/min). The simulation outputs on NPD (%) for different droplet sizes are linearly correlated with an average Pearson’s correlation coefficient of 0.98 for 30 L/min, 0.93 for 55 L/min, and 0.95 for 85 L/min. Subsequent check of the slope *m* for the linear best-fit trendline, through the scatter plots of RANS and LES-based NPD data, indicates how similar the estimates are quantitatively; the mean measures therein being $m = 1.113$ for 30 L/min, $m = 1.052$ for 55 L/min, and $m = 1.177$ for 85 L/min; with the value 1 signifying exact equivalence. The statistical operations were carried out on Mathematica 12.0 (Wolfram Research, Champaign, Illinois).

Also at this point, to think of a realistic exposure to a COVID-19 carrier: the vulnerable individual can be considered to inhale at different airflow rates over the duration of exposure. In such context, Panel A in Fig. 5 extracts the averaged nasopharyngeal deposition for the different tested inhalation rates in the two test subjects. Such inhalation-averaged transmission presents an approximate dehydrated droplet size range of 2.5–15.0 $\upmu$, for a minimum 2% NPD for each droplet size.

### Droplets that are better at carrying the virions

The next pertinent question is: *how effective are these droplets at carrying virions?* SARS-CoV-2 belongs to a diverse family of single-stranded RNA viruses^44,46^, and as noted before, virological assessments^14^ done on the sputum of hospitalized COVID-19 patients show an averaged viral load of $7.0 \times 10^6$ RNA copies/mL of oral fluid, with the peak load being $2.35 \times 10^9$ copies/mL. For the average load, simple calculations (see “Assessing the probability for at least 1 virion being embedded on a respiratory ejecta droplet emanating from an infected individual” in the methods) show that the probability that a dehydrated 10-$\upmu$ droplet (contracted from its original size of $\sim \,33.33$ $\upmu$) will carry at least 1 virion is 13.6%. The same number is 45.8% for a post-shrinkage 15-$\upmu$ droplet (contracted from its original size of $\sim 50$ $\upmu$). The probability drops exponentially to 0.2% for a 2.5-$\upmu$ dehydrated droplet (contracted from its original size of $\sim 8.33$ $\upmu$). Now, with existing data on the size distribution of expelled droplets during normal speaking (see Panel B, Fig. 5), the proportion of virion deposits at the nasopharynx by different droplet sizes can be computed (see Panel C, Fig. 5) by using the transmission data presented in Fig. 3, coupled with the mathematical framework laid out in “Estimating the number of virions transmitted to the nasopharynx”. The virion deposition trends are again from droplets that are being inhaled post-dehydration.

**Figure 6:**
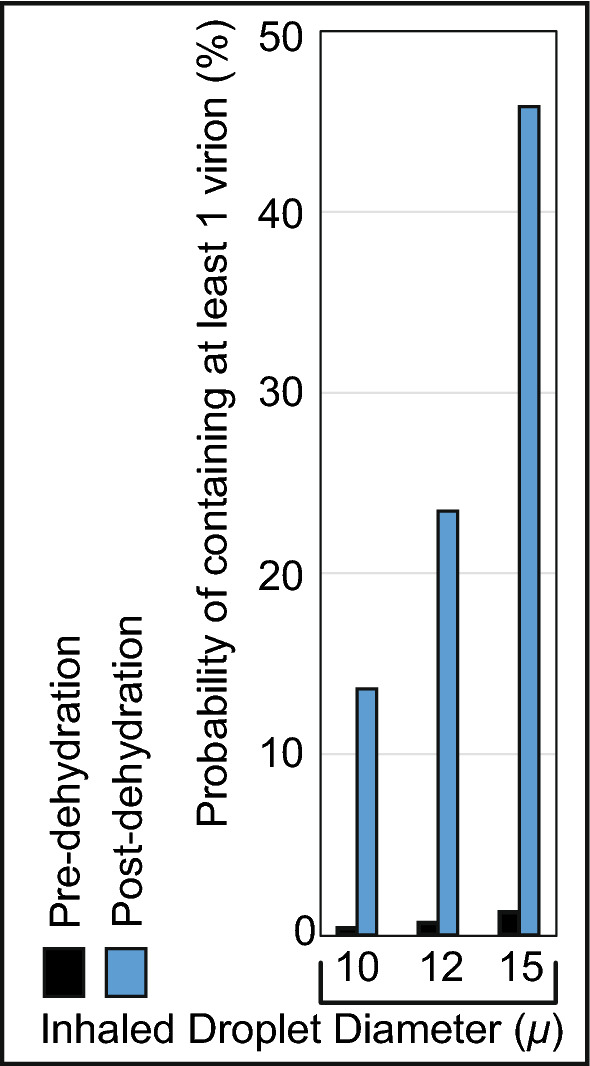
Effect of environmental dehydration assessed via probabilistic interpretation of a droplet to contain at least 1 virion, based on whether the droplet size at inhalation is pre-dehydration or post-dehydration. The plots are generated using Microsoft Excel 365; link to software homepage.

Significantly enough: in the absence of environmental dehydration, the probability of 1 virion being embedded in, for instance, a 10-$\upmu$ droplet plummets to 0.37% (see Fig. 6). This rationalizes why in geographic regions with high humidity (and hence relatively less dehydration and shrinkage of respiratory ejecta), the pandemic’s spread has been somewhat measured^47,48^.

### On the SARS-CoV-2 infectious dose

The *infectious dose* is a fundamental virological measure quantifying the number of virions that can go on to start an infection; the value of which is still not conclusively known for SARS-CoV-2^49^. Theoretically, according to the *independent action hypothesis*^50^, even a single virion can potentially establish an infection in highly susceptible systems. Whether the hypothesis is true for humans and specifically for SARS-CoV-2 transmission is as yet undetermined. The rapid spread of COVID-19 though a priori suggests a small infective dose for the disease, that is triggering inter-human transmission.

Since it would be unethical at this point to expose subjects to SARS-CoV-2 (especially in the absence of well-evidenced remediating therapeutics—as of February 2021), this study proposes a novel strategy synergizing computational tracking and virological data, to estimate the infectious dose. Based on the nasopharyngeal transmission trends (Fig. 3) and the virion transmission data (Panels B,C of Fig. 5), for a 5-min exposure: the number of virions depositing at the susceptible individual’s nasopharynx is 11, considering average RNA load in the carrier’s sputum. On the contrary, if the infecting individual is in the disease phase with peak RNA load, as many as 3835 virions will be deposited on the nasopharynx of the exposed individual over 5 min (see Panel D in Fig. 5).

To put the computed prediction on virion transmission into context, consider now the March 2020 Skagit Valley Chorale superspreading incident^5^, where a COVID-19 carrier infected 52 other individuals in a 61-member choir group. Exposure time there was reported to be 2.5 h. The subjects were positioned close to each other; which justifies discounting the effect of spatial ventilation for a conservative estimate of the number of virions that a susceptible individual would have been exposed to. Therefore, for an average RNA load (assuming that the carrier had mild-to-moderate symptoms), coupled with derived exposure estimates from previous paragraph, the number of virions depositing at a closely-situated individual’s nasopharynx over the 2.5-h duration approximates to: (11/5) virions per minute $\times$ 60 min $\times$ 2.5 h $= 330$. So, an order of $\mathcal {O}(10^2)$ could be reckoned as a conservative estimate for SARS-CoV-2’s infectious dose; the scale agreeing with preliminary estimates based on the viral replication rates^51^.

#### A review of projections for the SARS-CoV-2 infectious dose, as per other reports

A recent dose titration study^52^ of SARS-CoV-2 in a ferret model has shown 500 as the lower tested limit for the number of virions needed to launch an infection; the study extracted the dose in terms of the plaque-forming units (PFU)—which is a measure used in virology to describe the number of virus particles capable of forming plaques per unit volume. The quantitative estimate of the infectious dose, being of the order $\mathcal {O}(10^2)$, agrees with the findings presented here. Moreover, though not yet shown through experimental models in humans, multiple preliminary critiques^53,54^ expect the infectious dose of SARS-CoV-2 to be similar to that of other coronaviruses, such as SARS-CoV. According to a key publication^55^ from the 2010s, the SARS-CoV dose that correlated to 10% and 50% adverse responses (i.e., illness) was estimated respectively at 43 and 280 PFU. This confirms the feasibility of the infectious dose ($\approx 300$, or more conservatively $\mathcal {O}(10^2)$) derived here for SARS-CoV-2.

## Discussion

- *On the practicality of viral load used in the calculations*—Through tissue culture examinations for respiratory infections, it is fairly well recognized^56^ that only a small fraction of virions are actually able to infect a human cell, and that this fraction decreases rapidly with increasing duration from the time of initial infection of the carrier. So, the SARS-CoV-2 infectivity is being conjectured to peak well before the viral load reaches a maximum. This substantiates the use of averaged viral load (i.e. $7.0 \times 10^6$ RNA copies/mL) in the carrier’s sputum for the virological calculations, while deducing the conservative upper estimate for the SARS-CoV-2 infectious dose.
- *On the significance of the hazardous droplet sizes*—Whereas the computed data is post-processed to specifically extract the droplet sizes that tend to target the nasopharynx, a vastly larger remainder (comprising predominantly the droplets that are smaller than 5 $\upmu$) actually go further down the respiratory tract (considering that the air passageways narrow down to just a few microns in the lower airway). However, the significantly pronounced surface area of the lower airspaces, together with the relative scarcity of ACE2 receptors there, validates the robustness of the modeling approach i.e. focusing on the droplets that deposit on the ACE2-rich epithelial cells at the nasopharynx. Also, the probability of droplets smaller than 5 $\upmu$ to carry a virion is often trivial; e.g. the probability of containing a virion is only 1.7% for a 5-$\upmu$ dehydrated droplet; for the related analytical framework, see “Assessing the probability for at least 1 virion being embedded on a respiratory ejecta droplet emanating from an infected individual”.
- *On the number of simulated droplets*—The reader should note that the modeling framework to track approximately 3000 monodispersed droplets of discrete sizes is a mathematical idealization employed to obtain statistically robust data on the intranasal droplet transmission trends for each size and identify the specific size range for which a higher fraction of the inhaled droplets would land directly at the infection-prone nasopharynx in the upper respiratory pathway. Hence, the focus was not on replicating a realistic quantity and size distribution of droplets, which would be functions of a wide range of environmental and locational factors^57^, such as wind and inter-individual distances.
- *On the “trap” boundary condition in the simulations*—The numerical scheme used in this study tracks the inhaled droplets as long as they are airborne inside the intranasal space and stops the tracking when the droplets land on the airway tissue surfaces, which have a no-slip boundary condition. In other words, the droplet trajectories would cease once they touch a wall and the effects of any possible accretion and erosion of droplets at the walls were not considered. The feasibility of such entrapment is backed by the physicochemical properties^58^ of mucus which washes the upper airway walls. Mucus in the nose exhibits^59^ a pH range of 5.5–6.5. Mucins, which are the main component of the mucus secretions from goblet and epithelial cells, are known for inducing drastically different rheological and mechanical properties to mucus based on several ambient factors, of which the dominant one is the medium pH. The pH range in the nasal mucus is associated^60^ with a distinct hydrophilic behavior, which justifies the assumption of the airway walls to be perfectly absorbing via surface tension forces. The absorbed droplets will spread on impact^61–64^, and mechanistically, the virions embedded in the droplets could be conjectured to disperse over and percolate through the top gel-like layer of mucus.
  Additionally, note that this study considers a droplet to “land” on the airway walls, only when the droplet center enters the layer of mesh cells that adhere to the walls. To enhance accuracy of tracking, the mesh at the airway-tissue interfaces comprises three layers of prism cells, with the thickness of each layer being approximately 30 $\upmu$. With similar mesh refinement, the use of the entrapment boundary condition at the walls during nasal transport is also supported by a long history of publications on particle and droplet tracking by multiple research groups^65–68^, along with several such studies^43,69^ presenting a strong agreement between CFD simulations and in vitro experiments.
- *On the limitations of the virion exposure estimate*—Spatiotemporal parameters, such as ambient airflow and ventilation rates; and subject-specific biological variables, such as innate immune response and anthropometric differences, are only a subset of the many critical factors that can have a strong correlation on quantifying viral exposure and infectious dose, and as such, the estimates for virion transmission presented here has not been substantiated by an epidemiological model. To that end, obtaining an exact and conclusive measure of the SARS-CoV-2 infectious dose will require a wide in vivo study. The cross-disciplinary strategy presented here (i.e. integration of numerical simulations of transport in complex anatomic pathways with virological assessments and respiratory ejecta data) could however be potentially extended as a sub-component of a full-scale epidemiological model, for an exact quantification of virologic parameters, such as the infectious dose.
- *On the continuum assumption related to the ejecta generation from oral liquids*—The mathematical approach for the estimation of virion contamination in the respiratory ejecta has, by the very nature of it, presumed a simplistic estimate of viral load in the ejected droplets, based on a continuum-based argument that the spatial distribution of virions could be considered uniform in the sputum. In reality, how the complex rheology of oral fluids might affect the ejecta generation and subsequent break-down^70^, and the resultant volumetric concentration of virions embedded in the expiratory remnants—are also critical open questions.
- *On the size of the test cohort*—This study is somewhat limited by the small sample size, primarily owing to the lack of CT scans in subjects with otherwise disease-free airways. To get a realistic insight on the intranasal transport phenomena at the onset of any respiratory infection, it is preferred that we base the in silico cavity reconstructions on CT-normal images. Nonetheless, the preliminary findings presented here could be considered an important step in the mechanistic characterization of the transmission dynamics for inhaled pathogens, such as SARS-CoV-2.

## The main takeaways

To highlight the main finding from this study: the detection of the inhaled droplet sizes ($\sim$ 2.5 to 19 $\upmu$, see “Droplet size range that targets the nasopharynx”) that specifically target the infection-prone nasopharynx, can provide a pivotal resource in mitigating the pandemic. For instance, the information on the droplet sizes that tend to launch the initial infection at the nasopharynx could be utilized to inform public policy on social distancing and in the design^71–74^ of novel masks and face-coverings that can execute targeted screening of only the hazardous droplet sizes and in the process, be more breathable and user-friendly than the mask respirators available now. The findings can additionally extend salient inputs to the mechanistic design of topical anti-viral therapeutics^75–78^ and targeted intranasal vaccines^79–83^, that could be tailored to undergo regional deposition directly over the infected nasopharynx, thereby significantly pronouncing their therapeutic indices, especially when compared to systemically administered vaccines and other prophylactics.

Obtained as a corollary, the $\mathcal {O}(10^2)$ estimate for the SARS-CoV-2 infectious dose (see “On the SARS-CoV-2 infectious dose”) is also noteworthy. The low order underlines the high communicability of this disease, especially if discerned in the perspective of other airborne transmissions, e.g. the infective dose for influenza A virus, when administered through aerosols to human subjects lacking serum neutralizing antibodies, is at least an order greater and ranges between 1950 and 3000 virions^84^.

## Data Availability

This project has generated simulated, quantitative, de-identified data on regional deposition over nasal tissues. The digitized anatomic geometries, the simulation data-sets (including Fluent .cas and .dat files), and the numeric protocols; along with MATLAB codes, Wolfram Mathematica notebooks, and Microsoft Excel spreadsheets used for data post-processing—are available on-request from the author, through a shared-access Google Drive folder^85^.

## References

[1] Johns-Hopkins-University. Coronavirus Resource Center. Web link. Accessed 28 Feb 2021.

[2] Bourouiba L (2020). Turbulent gas clouds and respiratory pathogen emissions: Potential implications for reducing transmission of COVID-19. JAMA.

[3] Mittal R, Ni R, Seo JH (2020). The flow physics of COVID-19. J. Fluid Mech..

[4] Dyal JW (2020). COVID-19 among workers in meat and poultry processing facilities—19 States, April 2020. Morbidity Mortality Wkly. Rep..

[5] Miller SL (2020). Transmission of SARS-CoV-2 by inhalation of respiratory aerosol in the Skagit Valley Chorale superspreading event. Indoor Air.

[6] Li W (2020). Characteristics of household transmission of COVID-19. Clin. Infect. Dis..

[7] Xie X, Li Y, Sun H, Liu L (2009). Exhaled droplets due to talking and coughing. J. R. Soc. Interface.

[8] Wells WF (1934). On airborne infection: Study II, droplets and droplet nuclei. Ame. J. Epidemiol..

[9] Xie X, Li Y, Chwang ATY, Ho PL, Seto WH (2007). How far droplets can move in indoor environments—revisiting the Wells evaporation-falling curve. Indoor Air.

[10] Resnick, B. The debate over “airborne” coronavirus spread, explained. Web link. Accessed 20 Feb 2021.

[11] Hou YJ (2020). SARS-CoV-2 reverse genetics reveals a variable infection gradient in the respiratory tract. Cell.

[12] Matheson NJ, Lehner PJ (2020). How does SARS-CoV-2 cause COVID-19?. Science.

[13] Dickson RP, Erb-Downward JR, Martinez FJ, Huffnagle GB (2016). The microbiome and the respiratory tract. Annu. Rev. Physiol..

[14] Wölfel R (2020). Virological assessment of hospitalized patients with COVID-2019. Nature.

[15] Basu S, Chakravarty A (2020). From SARS-CoV-2 infection to COVID-19 disease: A proposed mechanism for viral spread to the lower airway based on in silico estimation of virion flow rates. medRxiv.

[16] Patel MR (2020). Performance of oropharyngeal swab testing compared to nasopharyngeal swab testing for diagnosis of COVID-19. Clin. Infect. Dis..

[17] Stadnytskyi V, Bax CE, Bax A, Anfinrud P (2020). The airborne lifetime of small speech droplets and their potential importance in SARS-CoV-2 transmission. Proc. Natl. Acad. Sci..

[18] Basu S, Kabi P, Chaudhuri S, Saha A (2020). Insights on drying and precipitation dynamics of respiratory droplets from the perspective of COVID-19. Phys. Fluids.

[19] Bar-On YM, Flamholz A, Phillips R, Milo R (2020). Science forum: SARS-CoV-2 (COVID-19) by the numbers. Elife.

[20] Garcia GJM (2009). Dosimetry of nasal uptake of water-soluble and reactive gases: A first study of interhuman variability. Inhalation Toxicol..

[21] He X, Reponen T, McKay RT, Grinshpun SA (2013). Effect of particle size on the performance of an N95 filtering facepiece respirator and a surgical mask at various breathing conditions. Aerosol Sci. Technol..

[22] Frank-Ito DO, Wofford M, Schroeter JD, Kimbell JS (2016). Influence of mesh density on airflow and particle deposition in sinonasal airway modeling. J. Aerosol Med. Pulm. Drug Deliv..

[23] Basu S, Witten N, Kimbell JS (2017). Influence of localized mesh refinement on numerical simulations of post-surgical sinonasal airflow. J. Aerosol Med. Pulmonary Drug Deliv..

[24] Inthavong K (2019). Geometry and airflow dynamics analysis in the nasal cavity during inhalation. Clin. Biomech..

[25] Zhang Y (2019). Computational investigation of dust mite allergens in a realistic human nasal cavity. Inhalation Toxicol..

[26] Basu S, Frank-Ito DO, Kimbell JS (2018). On computational fluid dynamics models for sinonasal drug transport: Relevance of nozzle subtraction and nasal vestibular dilation. Int. J. Numer. Methods Biomed. Eng..

[27] Farzal Z (2019). Comparative study of simulated nebulized and spray particle deposition in chronic rhinosinusitis patients. Int. Forum Allergy Rhinol..

[28] Kimbell JS (2019). Upper airway reconstruction using long-range optical coherence tomography: Effects of airway curvature on airflow resistance. Lasers Surg. Med..

[29] Kiaee M, Wachtel H, Noga ML, Martin AR, Finlay WH (2018). Regional deposition of nasal sprays in adults: A wide ranging computational study. Int. J. Numer. Methods Biomed. Eng..

[30] Brandon BM (2018). Comparison of airflow between spreader grafts and butterfly grafts using computational flow dynamics in a cadaveric model. JAMA Facial Plastic Surg..

[31] Brandon BM (2020). Nasal airflow changes with bioabsorbable implant, butterfly, and spreader grafts. Laryngoscope.

[32] Zhao K, Scherer PW, Hajiloo SA, Dalton P (2004). Effect of anatomy on human nasal air flow and odorant transport patterns: Implications for olfaction. Chem. Sens..

[33] Xi J, Longest PW (2008). Numerical predictions of submicrometer aerosol deposition in the nasal cavity using a novel drift flux approach. Int. J. Heat Mass Transf..

[34] Shanley KT, Zamankhan P, Ahmadi G, Hopke PK, Cheng YS (2008). Numerical simulations investigating the regional and overall deposition efficiency of the human nasal cavity. Inhalation Toxicol..

[35] Kelly JT, Prasad AK, Wexler AS (2000). Detailed flow patterns in the nasal cavity. J. Appl. Physiol..

[36] Longest PW, Vinchurkar S (2007). Validating CFD predictions of respiratory aerosol deposition: Effects of upstream transition and turbulence. J. Biomech..

[37] Perkins EL (2018). Ideal particle sizes for inhaled steroids targeting vocal granulomas: Preliminary study using computational fluid dynamics. Otolaryngol. Head Neck Surg..

[38] Tracy LF (2019). Impact of endoscopic craniofacial resection on simulated nasal airflow and heat transport. Int. Forum Allergy Rhinol..

[39] Hosseini S (2020). Use of anatomically-accurate 3-dimensional nasal airway models of adult human subjects in a novel methodology to identify and evaluate the internal nasal valve. Comput. Biol. Med..

[40] Doorly DJ, Taylor DJ, Schroter RC (2008). Mechanics of airflow in the human nasal airways. Respir. Physiol. Neurobiol..

[41] Baghernezhad N, Abouali O (2010). Different SGS models in Large Eddy Simulation of 90 degree square cross-section bends. J. Turbulence.

[42] Ghahramani E, Abouali O, Emdad H, Ahmadi G (2017). Numerical investigation of turbulent airflow and microparticle deposition in a realistic model of human upper airway using LES. Comput. Fluids.

[43] Basu S (2020). Numerical evaluation of spray position for improved nasal drug delivery. Sci. Rep..

[44] V’kovski P, Kratzel A, Steiner S, Stalder H, Thiel V (2020). Coronavirus biology and replication: Implications for SARS-CoV-2. Nat. Rev. Microbiol..

[45] Finlay WH (2001). The Mechanics of Inhaled Pharmaceutical Aerosols: An Introduction.

[46] Moreno-Eutimio MA, López-Macías C, Pastelin-Palacios R (2020). Bioinformatic analysis and identification of single-stranded RNA sequences recognized by TLR7/8 in the SARS-CoV-2, SARS-CoV, and MERS-CoV genomes. Microbes Infect..

[47] Wang J, Tang K, Feng K, Lv W (2020). High temperature and high humidity reduce the transmission of COVID-19. SSRN.

[48] Wang M (2020). Temperature significantly changed COVID-19 transmission in 429 cities. medRxiv.

[49] Lakdawala, S. & Gaglia, M. What we do and do not know about COVID-19’s infectious dose and viral loads. Web link. Accessed 20 Feb 2021.

[50] Zwart MP (2009). An experimental test of the independent action hypothesis in virus-insect pathosystems. Proc. R. Soc. B Biol. Sci..

[51] Wang D (2020). Population bottlenecks and intra-host evolution during human-to-human transmission of SARS-CoV-2. bioRxiv.

[52] Ryan KA (2020). Dose-dependent response to infection with SARS-CoV-2 in the ferret model: Evidence of protection to re-challenge. bioRxiv.

[53] Brosseau LM, Roy CJ, Osterholm MT (2020). Facial masking for COVID-19. N. Engl. J. Med..

[54] Geddes, L. Does a high viral load or infectious dose make COVID-19 worse? Web link. Accessed 20 Feb 2021.

[55] Watanabe T, Bartrand TA, Weir MH, Omura T, Haas CN (2010). Development of a dose-response model for SARS coronavirus. Risk Anal. Int. J..

[56] Brooke CB (2013). Most influenza A virions fail to express at least one essential viral protein. J. Virol..

[57] Li H (2020). Dispersion of evaporating cough droplets in tropical outdoor environment. Phys. Fluids.

[58] Leal J, Smyth HDC, Ghosh D (2017). Physicochemical properties of mucus and their impact on transmucosal drug delivery. Int. J. Pharm..

[59] Beule AG (2010). Physiology and pathophysiology of respiratory mucosa of the nose and the paranasal sinuses. GMS Curr. Top. Otorhinolaryngol. Head Neck Surg..

[60] Sumarokova M (2018). Influencing the adhesion properties and wettability of mucin protein films by variation of the environmental pH. Sci. Rep..

[61] Nath S, Quéré D (2020). Spreading of viscous drops on a liquid-infused solid. Bull. Am. Phys. Soc..

[62] Basu S, Yawar A, Concha A, Bandi MM (2015). Modeling drop impacts on inclined flowing soap films. APS Div. Fluid Dyn. Meet. Abstr..

[63] Yawar A, Basu S, Concha A, Bandi MM (2015). Experimental study of drop impacts on soap films. APS Div. Fluid Dyn. Meet. Abstr..

[64] Basu S, Yawar A, Concha A, Bandi MM (2017). On angled bounce-off impact of a drop impinging on a flowing soap film. Fluid Dyn. Res..

[65] Dastan A, Abouali O, Ahmadi G (2014). CFD simulation of total and regional fiber deposition in human nasal cavities. J. Aerosol Sci..

[66] Schroeter JD, Tewksbury EW, Wong BA, Kimbell JS (2015). Experimental measurements and computational predictions of regional particle deposition in a sectional nasal model. J. Aerosol Med. Pulm. Drug Deliv..

[67] Inthavong K, Tian ZF, Tu JY, Yang W, Xue C (2008). Optimising nasal spray parameters for efficient drug delivery using computational fluid dynamics. Comput. Biol. Med..

[68] Inthavong K, Ge Q, Se CMK, Yang W, Tu JY (2011). Simulation of sprayed particle deposition in a human nasal cavity including a nasal spray device. J. Aerosol Sci..

[69] Schroeter JD, Garcia GJM, Kimbell JS (2011). Effects of surface smoothness on inertial particle deposition in human nasal models. J. Aerosol Sci..

[70] Abkarian M, Mendez S, Xue N, Yang F, Stone HA (2020). Speech can produce jet-like transport relevant to asymptomatic spreading of virus. Proc. Natl. Acad. Sci..

[71] Chakraborty A (2020). Simulating inhaled transport through bio-inspired pathways in mask filters. Bull. Am. Phys. Soc..

[72] Yuk J (2020). 3D-printing mask filters inspired by animal nasal cavity. Bull. Am. Phys. Soc..

[73] Chung C (2020). Vortex traps to capture particles with reduced pressure loss in respiratory masks. Bull. Am. Phys. Soc..

[74] Yuk J (2021). Bio-inspired mask filters with breathing resistance control. Bull. Am. Phys. Soc..

[75] Higgins TS (2020). Intranasal antiviral drug delivery and coronavirus disease 2019 (COVID-19): A state of the art review. Otolaryngol. Head Neck Surg..

[76] Ferrer G, Westover J (2020). In vitro virucidal effect of intranasally delivered chlorpheniramine maleate compound against severe acute respiratory syndrome coronavirus 2 (SARS-CoV-2). Res. Square.

[77] Xiong R (2020). Novel and potent inhibitors targeting DHODH, a rate-limiting enzyme in de novo pyrimidine biosynthesis, are broad-spectrum antiviral against RNA viruses including newly emerged coronavirus SARS-CoV-2. bioRxiv.

[78] Lao, Y. *et al.* Identifying the optimal parameters for sprayed and inhaled drug particulates for intranasal targeting of SARS-CoV-2 infection sites. arXiv:2010.16325 **(preprint)** (2020).

[79] Oberdick, J. Rethinking the traditional vaccine delivery in response to coronaviruses. Web link. Accessed 20 Feb 2021.

[80] Estep, P. *et al.* SARS-CoV-2 (2019-nCoV) vaccine. White Paper link. Accessed 30 Sep 2020.

[81] Kim MH, Kim HJ, Chang J (2019). Superior immune responses induced by intranasal immunization with recombinant adenovirus-based vaccine expressing full-length Spike protein of Middle East respiratory syndrome coronavirus. PLoS One.

[82] WebMD. Nasal spray COVID vaccine shows promise in animal trials. Web link. Accessed 20 Feb 2021.

[83] Rohaim MA, Munir M (2020). A scalable topical vectored vaccine candidate against SARS-CoV-2. Vaccines.

[84] Nikitin N, Petrova E, Trifonova E, Karpova O (2014). Influenza virus aerosols in the air and their infectiousness. Adv. Virol..

[85] Basu, S. COVID-19 anatomic CFD data. Link to Google Drive folder (2021).

